# Prenatal diagnosis of sirenomelia with anencephaly and craniorachischisis totalis: A case report study

**DOI:** 10.1097/MD.0000000000009020

**Published:** 2017-12-15

**Authors:** Charalampos Theofanakis, Marianna Theodora, Michail Sindos, George Daskalakis

**Affiliations:** 1st Department of Obstetrics & Gynecology, National & Kapodistrian University of Athens, Alexandra Hospital, Athens, Greece.

**Keywords:** anencephaly, craniorachischisis totalis, oligohydramnios, sirenomelia

## Abstract

**Rationale:**

Sirenomelia and anencephaly are well-defined congenital malformations that usually occur independently.

**Patient concerns:**

We report a case of combined sirenomelia, anencephaly and complete rachischisis, diagnosed in the 16th week of gestation.

**Diagnoses:**

To our knowledge, this is the 7th case in the literature and the first that is diagnosed so early in pregnancy.

**Interventions:**

The final diagnosis is confirmed with radiological examination after the termination of pregnancy.

**Outcomes:**

Prenatal diagnosis of sirenomelia is difficult due to the presence of kidney agenesis and severe oligohydramnios.

**Lessons:**

The combination of sirenomelia and craniorachischisis totalis is extremely rare and prenatal ultrasound scan are a challenge, even for experts in the field.

## 1 Introduction

Sirenomelia sequence, also known as sirenomelia, is a birth defect of the lower body, characterized by apparent fusion of the legs into a single lower limb.^[1]^ It is a multisystemic condition associated with severe visceral abnormalities, especially of urogenital and gastrointestinal origin. It presents with a variety of phenotypes and is incompatible with life, while death usually occurs in the perinatal period.^[2,3]^ The incidence of sirenomelia varies between 1.1 and 4.2 per 100,000 births and has no difference among ethnic groups.^[4]^ It is diagnosed by prenatal ultrasonography, while antenatal ultrasonography scan findings include oligohydramnios, renal agenesis, and a fibula between the tibiae.^[5]^

Anencephaly is characterized by an open neural tube in the cephalic region, with an exposed mass of degenerating neural tissue on the skull floor. The cranial vault is absent, producing characteristic bulging of the eyes and absence of the neck. It is presented with a gross malformation in both the membranous neurocranium and chondrocranium. Anencephaly is classified as meroacrania if the defect does not affect the foramen magnum, holoacrania if it extends through the foramen magnum, and holoacrania with rachischisis if it presents with spina bifida.^[6]^

The coexistence of sirenomelia with anencephaly and rachischisis totalis is a very rare condition and there have been only 6 cases reported in the literature so far.

## 2 Case report

A 20-year-old woman with an uncomplicated previous pregnancy and an unremarkable medical and family history was first seen at 25 weeks of her second gestation. During ultrasound scan, severe oligohydramnios and growth restriction were noted and she was referred to our department for investigation and management of the pregnancy.

**Figure 1:**
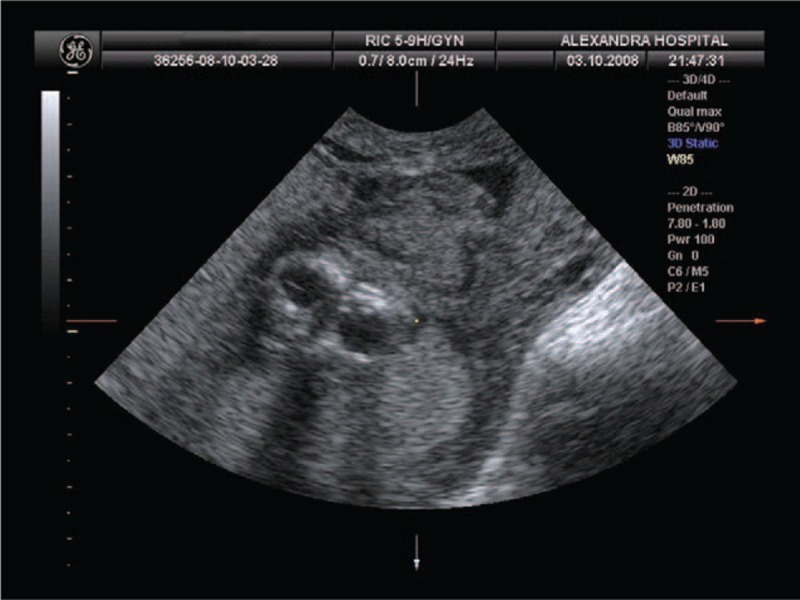
Second trimester ultrasonographic image of an anencephalic fetus.

**Figure 2:**
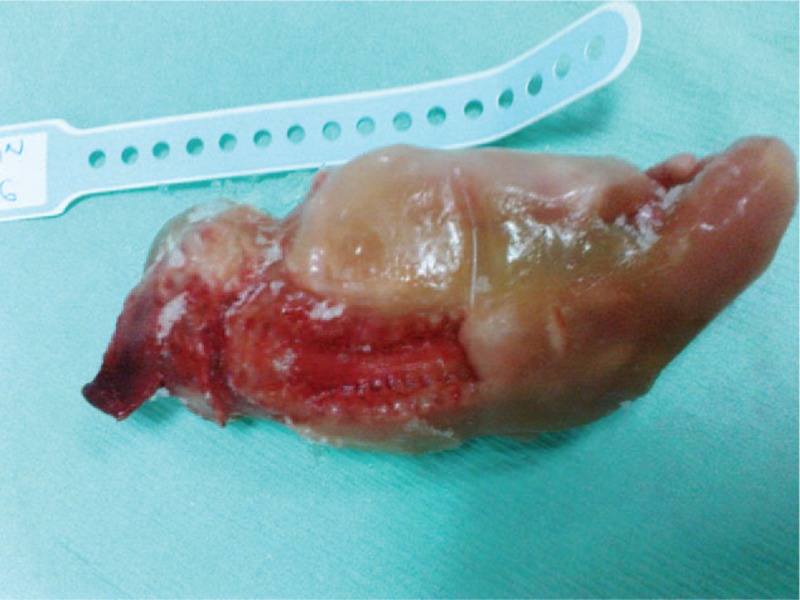
Fetus with sirenomelia, anencephaly, and craniorachischisis totalis shortly after the termination of pregnancy.

Ultrasonography demonstrated anencephaly (Fig. 1) and spina bifida. Additionally, a single femoral bone was identified during the examination. Due to severe oligohydramnio, fetal anatomy was assessed with difficulty. Based on the measurements of the femoral bone, the gestational age was 16 weeks. Pregnancy termination was decided due to anencephaly and informed consent was given. External examination of the fetus after the termination of the pregnancy showed anencephaly with complete rachischisis, absence of external genitalia, imperforate anus, and only 1 lower extremity (Fig. 2).

Internal examination showed the absence of the rectum and both kidneys, ureters, bladder, and urethra. The diagnosis of anencephaly and symelia apus was done both with external examination and radiological examination of the fetus.

## 3 Discussion

Sirenomelia with anencephaly was first reported in 1953 by Battaglia and Fraccaro.^[7]^ In 1991, Rodriguez et al,^[8]^ described a case of sirenomelia combined with anencephaly, on a monozygotic twin. Ultrasound scan at 25 weeks of gestation demonstrated 1 normal twin and 1 with alteration of the head and abnormal lower limbs. The twins were delivered at 32 weeks of gestation. The first twin was a female who underwent laparotomy and right adnexectomy right after birth due to a large adnexal cyst that proved to be a mature cystic teratoma with granulomatous reaction against squamous epithelial cells in the wall of the cyst. An autopsy report showed a duplication of the cervix, uterus, and vagina and a normal left ovary. The second twin was a hydropic stillborn anencephalic sireniform fetus of unidentifiable sex. External examination showed the absence of cranial vault, bulging eyes, cleft palate, flat nose, low set and malformed ears, along with a short neck.^[8]^

In our case reported, the patient had a singleton pregnancy that was diagnosed at her first visit for an ultrasound scan at 25 weeks of gestation. The ultrasound showed severe oligohydramnios and intrauterine growth restriction (IUGR). Upper limbs appeared to be normal while only 1 femoral bone was recognized. Our presented case combines cephalic and caudal defects of the embryo and could be considered an example of the “axial mesodermal dysplasia spectrum.”

The originality of this case lies on the fact that during the first prenatal visit, in spite of severe oligohydramnios, we were able to identify via sonography in the early second trimester of pregnancy, findings that constituted with anencephaly and sirenomelia.

Anencephaly in patients with sirenomelia could be the only neural tube defect (NTD) or it could be associated with an exposed area of spinal cord of variable extent. The extent of the NTD could be inversely correlated with the lower limb abnormalities.^[9]^ Although our case was a singleton pregnancy, sirenomelia combined with anencephaly seem to be in excess frequency in monozygotic twins.^[10]^

Polyhydramnios is found frequently in gestations of anencephalic infants while oligohydramnios is observed mostly in sirenomelia.^[9]^ In our case, the patient had oligohydramnios, which made the prenatal diagnosis a difficult process. However, based on the ultrasound findings of severe IUGR, single femoral bone, and anencephaly, the pregnancy was terminated and the diagnosis was confirmed by observational and radiologic studies of the fetus. Ultrasonographic diagnosis of anencephaly could be easy but the combination of anencephaly and oligohydramnios should set the suspicion for more complex anomalies. Prenatal diagnosis of sirenomelia is difficult due to the presence of kidney agenesis and severe oligohydramnios. The final diagnosis is confirmed with radiological examination after the termination of pregnancy.

## 4 Conclusions

Sirenomelia with anencephaly and rachischisis totalis is a rare complication of pregnancy. This anomaly is not compatible with life and the diagnosis is extremely challenging, thus highlighting the importance of a thorough ultrasound examination. Termination of pregnancy is recommended upon diagnosis, with a detailed consultation followed by close-up monitoring for future pregnancies.
